# Current state and promise of user-centered design to harness explainable AI in clinical decision-support systems for patients with CNS tumors

**DOI:** 10.3389/fradi.2024.1433457

**Published:** 2025-01-13

**Authors:** Eric W. Prince, David M. Mirsky, Todd C. Hankinson, Carsten Görg

**Affiliations:** ^1^ Department of Neurosurgery, University of Colorado School of Medicine, Aurora, CO, United States; ^2^ Department of Biostatistics & Informatics, Colorado School of Public Health, Aurora, CO, United States; ^3^ Morgan Adams Foundation Pediatric Brain Tumor Research Program, University of Colorado School of Medicine, Aurora, CO, United States; ^4^ Department of Radiology, University of Colorado School of Medicine, Aurora, CO, United States

**Keywords:** explainable artificial intelligence (XAI), user-centered design (UCD), clinical neuro-oncology, MR imaging, clinical decision support

## Abstract

In neuro-oncology, MR imaging is crucial for obtaining detailed brain images to identify neoplasms, plan treatment, guide surgical intervention, and monitor the tumor's response. Recent AI advances in neuroimaging have promising applications in neuro-oncology, including guiding clinical decisions and improving patient management. However, the lack of clarity on how AI arrives at predictions has hindered its clinical translation. Explainable AI (XAI) methods aim to improve trustworthiness and informativeness, but their success depends on considering end-users’ (clinicians') specific context and preferences. User-Centered Design (UCD) prioritizes user needs in an iterative design process, involving users throughout, providing an opportunity to design XAI systems tailored to clinical neuro-oncology. This review focuses on the intersection of MR imaging interpretation for neuro-oncology patient management, explainable AI for clinical decision support, and user-centered design. We provide a resource that organizes the necessary concepts, including design and evaluation, clinical translation, user experience and efficiency enhancement, and AI for improved clinical outcomes in neuro-oncology patient management. We discuss the importance of multi-disciplinary skills and user-centered design in creating successful neuro-oncology AI systems. We also discuss how explainable AI tools, embedded in a human-centered decision-making process and different from fully automated solutions, can potentially enhance clinician performance. Following UCD principles to build trust, minimize errors and bias, and create adaptable software has the promise of meeting the needs and expectations of healthcare professionals.

## 1 Introduction

In neuro-oncology, MR imaging is an essential component of patient management. It provides high-resolution, detailed images of the brain for precise identification and characterization of neoplasms, aids in formulating the most suitable therapeutic strategy, serves as a navigational aid for neurosurgeons during procedures, and is employed post-therapeutically to monitor the neoplasm's response to treatment and detect any early signs of recurrence. Patient management decisions require balancing potential benefits and risks. Effective coordination among team members ensures accurate data interpretation, streamlined workflows, and timely decision-making.

Recent progress in artificial intelligence (AI) related to neuroimaging makes the application to neuro-oncology timely and promising (1). AI technology can support healthcare professionals in making clinical decisions for neuro-oncology patients by providing evidence-based information and patient-specific tools (1). It thus promises to improve patient care by reducing diagnostic variation, preventing unnecessary treatments and surgeries, and decreasing associated healthcare costs (1, 2). Lack of clarity regarding how AI systems operate has been one of the reasons why the clinical translation of AI tools has been limited so far. Explainable AI (XAI) methods aim to produce explanations that improve trustworthiness, informativeness, confidence, fairness, and interactivity (3, 4). Given the varying criteria that constitute a good explanation, the success of XAI methods depends on considering the specific context and goals of end-users (clinicians) and their individual preferences (5). Additionally, clinical translation of XAI systems will require usable and familiar interfaces to clinicians (6).

User-Centered Design (UCD) is an iterative design process where designers prioritize user needs in every phase. It involves users throughout the process to create highly usable and accessible products. A UCD framework ensures that usability goals, user characteristics, environment, tasks, and workflow are given extensive attention at each stage. Previous studies on XAI to support MRI interpretation related to CNS tumors have lacked a direct and intentional focus (7). Theoretical solutions often adjust existing XAI solutions in a way that does not fit the clinical context. For instance, tasks like distinguishing between tumor tissue and healthy tissue or between brain metastases and glioma are already performed very well by humans (8–10). This leaves little room for AI to improve accuracy and makes it challenging to assess its impact due to small potential effect sizes and limited access to human experts (11). That does not imply that these lines of research should not be generally pursued. However, it does mean that the exact implementation context and expected value of the AI system being developed should be carefully considered. As such, the opportunity exists to design XAI systems tailored to clinical neuro-oncology (12).

There have been comprehensive reviews on the application of AI within clinical decision-making associated with neuro-oncological care (1, 12, 13). Voigtlaender et al. broadly discuss applying AI technology to clinical neurology practice (13). They note that advancements in AI technology point towards a variety of practical clinical neurology applications but that the clinical neurology community must engage in the development to ensure software adoptions, positive human-AI interactions, and improved clinical outcomes. Khalighi et al. survey the application of AI systems to clinical neuro-oncology specifically (1). Like Voigtlaender, they also point to several applications unique to clinical neuro-oncology, including CNS tumor diagnosis, determining prognosis, and treatment planning. In addition, they also stress the importance of engaging with all stakeholders to develop safe, ethical, and legally compliant AI software.

Familiar et al. provide a comprehensive assessment of the challenges in identifying and delineating subregions of pediatric brain tumors from MR images (14). Their goal is to use these subregions and their respective measurements to assess clinical response to treatment according to existing guidelines. This work implicitly represents an initial UCD effort in that a specific clinical task has been defined (i.e., response assessment from MR images) with particular areas for improvement that AI can potentially address. The next phase of such work is to design solutions to address these challenges and to evaluate their effectiveness. However, very few theoretical solutions have ever been assessed broadly in radiology (15).

It has been shown that AI assistance can have a variable impact on how humans make clinical decisions (16). Human experts may demonstrate automation bias or neglect, which causes them to overweigh or underweight values from AI predictions (11, 17). Human experts may also treat AI information in a manner that considers evidence from the AI and all other evidence as separate independent entities, and they do not predictably update their beliefs (11). There may also be biases that manifest over time after the AI system has been deployed in a setting; for example, a user may begin to preemptively predict AI responses as they interact with an AI system more (18).

Implementing a UCD approach during design and development ensures that AI systems are aligned with experts' needs and expectations. Chen and colleagues review the scientific literature on transparent AI for medical image analysis (12). After carefully assessing this literature, they define a set of clinical AI design guidelines that emphasize the importance of following a user-centered approach to designing, implementing, and evaluating AI for medical imaging analysis. This work is just one example framework; while others exist, it is important to consider the advantages and limitations of the framework chosen.

This review organizes various facets of applying XAI to support and enhance clinical decision-making within neuro-oncology. We specifically emphasize the intersection of the interpretation of MR images for neuro-oncology patient management decisions, XAI, and UCD. However, only some scientific studies have specifically evaluated AI-assisted radiological interpretation in CNS tumor care on a human-based application level. Therefore, we provide a resource that organizes the necessary concepts at a high level: (1) Design and Evaluation, (2) Translation, (3) Enhancing the User Experience and Efficiency, and (4) AI for Improved Clinical Outcomes in Neuro-Oncology Treatment.

## 2 Design and evaluation considerations regarding XAI in clinical neuro-oncology

**Figure 1:**
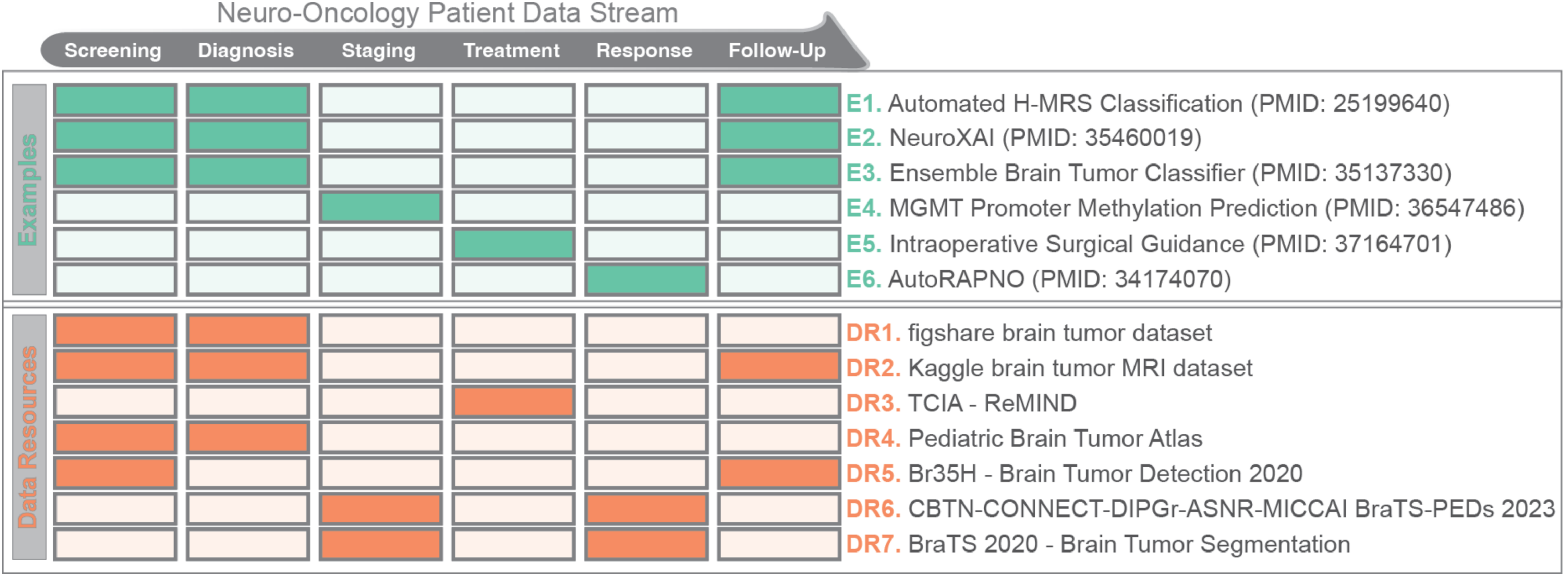
Stages in a patient data stream for neuro-oncology (top), along with example applications (green) and data resources (orange) for each stage. Tables 1, 2 provide additional details for the example applications and data resources (adapted from Kann et al.).

Clinical neuro-oncology relies on MR imaging in various contexts through the patient care trajectory. Figure 1 presents the data stream for neuro-oncology patients, including example applications and data resources for each stage. Other fields have developed a variety of UCD frameworks. Table 1 presents three examples from different fields and discusses their advantages and disadvantages.

**Table 1:** Description of user-centered design framework examples with advantages and disadvantages.

Name	Description	Advantages	Disadvantages
Design thinking (19)	Design thinking, originating from the industrial design community and popularized by Stanford's d.school, is a human-centric method for problem-solving across various fields. It consists of five stages: empathize, define, ideate, prototype, and test; it focusses on empathy, creativity, and iterative refinement	By centering on user needs and fostering teamwork, Design Thinking stimulates creative solutions. Constant testing and feedback enhance validation and reduce risk	Design thinking can be time-consuming and may incorporate biases. It may also overlook technical constraints and encounter resistance during organizational adoption, requiring customization to fit specific goals and needs
Nested model/extended nested model (20, 21)	The Nested Model, introduced by Munzner in the Information Visualization community, provides a methodical structure for crafting visualization systems. The Extended Nested Model for explainable AI is an expansion of the framework to enhance transparency and trust by concentrating on user-centric explanation factors	The Nested Model offers a systematic, in-depth approach to design, ensuring a deep understanding of user needs and problem areas. Its extension, the Extended Nested Model, boosts AI system transparency and trust, providing clearer user explanations for AI decisions and enhancing system efficacy	These structured design approaches can be complex and demand a delicate balance between transparency, efficiency, and user experience. They may also require supplementary ethical considerations to address broader impacts
INTRPRT (9)	Introduced within the biomedical community, the INTRPRT guidelines advocate for the development and evaluation of transparent ML systems, particularly in medical imaging. These guidelines stress the importance of balancing ML method development with evidence collection and considering human-centric design factors like format and interactivity. They also highlight the necessity of employing appropriate metrics and validating transparency techniques through a user-centered approach	These guidelines enhance transparency specifically in medical ML applications, promote evidence-based development, and focus on user-centered designs. They ensure relevance and effectiveness through correct metric usage and practical evaluation of transparency methods	Achieving transparency in ML systems can challenge the balance between performance and complexity, consume substantial computational resources, and face logistical hurdles in user validation. The guidelines may require continual updates to accommodate emerging techniques

**Table 2:** Example applications of AI within clinical care of patients with CNS tumors. IDs reflect entries in Figure 1.

ID	Clinical service	Application	Model	Description	PMID	Publication year
E1	Radiology	Classification of brain tumor from MRI	Laplacian Eigenmaps	The authors investigated whether nonlinear dimensionality reduction techniques improve unsupervised classification of brain tumor data compared to linear methods. They found that Laplacian eigenmaps (LE) combined with unsupervised clustering provided high accuracy in classifying glioma grade and distinguishing tumor and normal spectra, as well as achieving color-coded visualization of tumor and normal brain tissue	25199640	2015
E2	Radiology	Classification of brain tumor from MRI	Convolutional neural network	The NeuroXAI framework provides visualizations to increase the transparency and trust of deep learning models used in medical image analysis for brain imaging, making it a valuable tool for assisting radiologists and medical professionals in detecting and diagnosing brain tumors	35460019	2022
E3	Radiology	Classification of brain tumor from MRI	Ensemble model	A modified InceptionResNetV2 pre-trained model and an ensemble method combining InceptionResNetV2 and Random Forest Tree (RFT) are used to detect and classify brain tumors and their stages with high accuracy using brain MRI. The dataset size is augmented using C-GAN (Cyclic Generative Adversarial Networks)	35137330	2022
E4	Radiomics	Prediction of MGMT promoter methylation status	Convolutional neural network	This study proposes a deep learning-based approach using MR imaging data to identify methylation of the MGMT promoter in glioblastoma patients, which is a predictive biomarker of therapy response and prognosis, demonstrating comparable or better results than current methods with minimal parameters and incorporating an explainable AI analysis for clinical usability	36547486	2022
E5	Neurosurgery	Estimation of intraoperative brain shift for intraoperative surgical guidance	Convolutional neural network	This study used a convolutional neural network (CNN) to generate updated magnetic resonance images (uMR) to compensate for brain shifts during neurosurgeries. The CNN model was trained using preoperative MR (pMR) and intraoperative MR (iMR) images from 248 patients who underwent craniotomy for brain tumor removal. The system significantly reduced the target registration error (TRE), demonstrating the potential of using deep learning to correct brain shifts after dural opening	37164701	2023
E6	Radiology/Neuro-Oncology	Longitudinal response assessment in pediatric brain tumors	3-D U-net neural network	AutoRAPNO is an algorithm that automatically calculates the product of the 2D diameters of tumor segments, by searching for the largest tumor line segment and its perpendicular at each slice, applying criteria for measurable lesions, and selecting the largest product as the cross-sectional area, with the option to sum the top 4 products for multiple connected lesions and return them as the RAPNO score	34174070	2022

**Figure 2:**
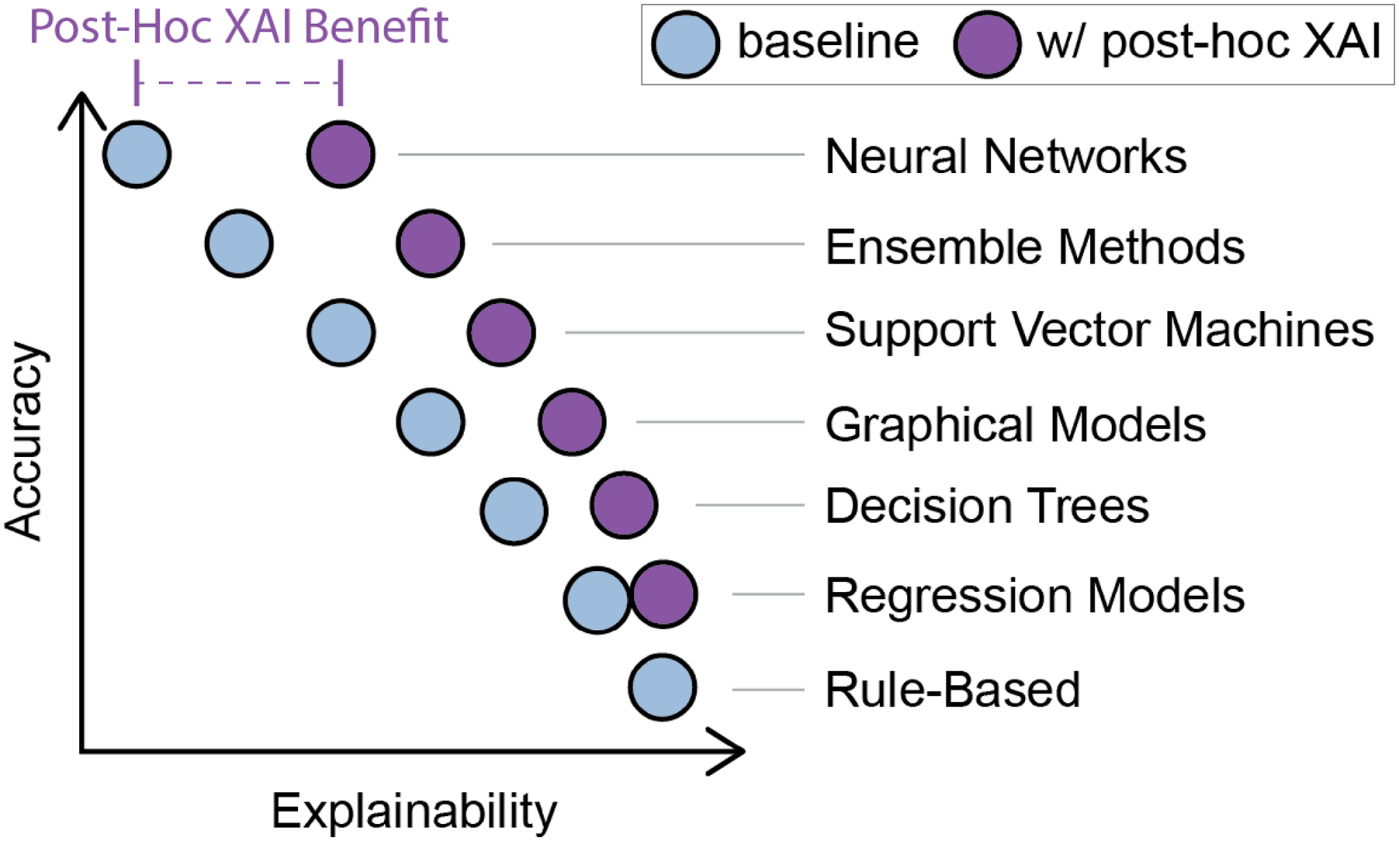
Accuracy vs. explainability trade-off in AI model types, with expected benefit from *post-hoc* XAI methods. Blue circles represent the tradeoff between accuracy (*y*-axis) and explainability (*x*-axis) concerning the listed XAI methods. The purple circles demonstrate how that trade-off shifts when using *post-hoc* XAI methods concerning each model type.

Because of the high risks associated with clinical decision-making, design requirements and specifications must be carefully defined to create an XAI interface. The design space for XAI visual representations is ample; examples include saliency maps, feature attribution maps, tabular data, and graphical networks. The implementation space for a model is also significant, including the type of model and its various parameters. Increasing the model's accuracy typically comes at the cost of decreasing explainability. Figure 2 illustrates the AI models' accuracy/explainability trade-off with expected *post-hoc* XAI benefits (explanations are generated after the model has been trained; see Section 3 for details). Neural networks are the most accurate but potentially expensive and typically have low explainability. Simple linear regression is less precise and flexible. However, regression models are directly interpretable because they have a clear, mathematical structure that allows for straightforward interpretation of the model's predictions. Other methods exist between these two extremes. Additional implementation considerations include choosing between single-modality data or multimodal data fusion methods and conducting the process on-premises or remotely.

When designing and evaluating AI tools to support clinical decision-making, it is crucial first to determine which UCD framework best suits your specific circumstances, such as team size and meeting frequency. Next, we must determine the details regarding patient care trajectories and clinical workflows within which the AI system will be deployed. By adhering to UCD principles, we can rigorously define the task specifications for the XAI interface by working directly with the target user groups. Finally, we can determine the specifications for the underlying AI models to optimize for factors like cost, time, and explainability. These pilot systems can then be evaluated in terms of clinical beneficence and usability via user studies before scaling to clinical settings. Because this process is iterative, the system can be constantly scrutinized and improved. Overall, UCD ensures that the software meets the expectations of providers, users, and affected patients and is aligned with software outputs and clinician expectations.

## 3 The role of XAI in translating AI into clinical neuro-oncology

Implementing AI in high-risk settings, such as the care of patients with CNS tumors, is hindered by what is known as the “black box” problem (22). The issue results from the computational complexity that underlies state-of-the-art AI systems and describes the inability of a user or programmer to identify the exact algorithmic process that yields the AI output. It is possible to create highly accurate predictive models. However, there are limits to our understanding of how the prediction was made and the associated uncertainty. Therefore, the translation of AI into clinical settings has been limited because of the ethical and legal need for clinicians to have a reliable understanding of the information they utilize in making patient care decisions and recommendations.

XAI methods broadly fall into two main categories: *ante hoc* and *post hoc*. *Ante hoc* methods involve the construction of AI models that are inherently interpretable but may sacrifice some degree of accuracy. On the other hand, *post hoc* methods generate explanations after the model has been trained.

Perturbation-based methods, such as LIME (Local Interpretable Model-agnostic Explanations) and SHAP (SHapley Additive exPlanations), are a commonly utilized class of *post hoc* XAI methods within biomedical research (23–26). These methods involve modifying inputs to observe the corresponding changes in the output, aiding in comprehending the importance of different features in the decision-making process. However, it's worth noting that these XAI methods are primarily tailored for AI engineers and researchers and may pose accessibility and interpretability challenges for clinicians (27).

**Figure 3:**
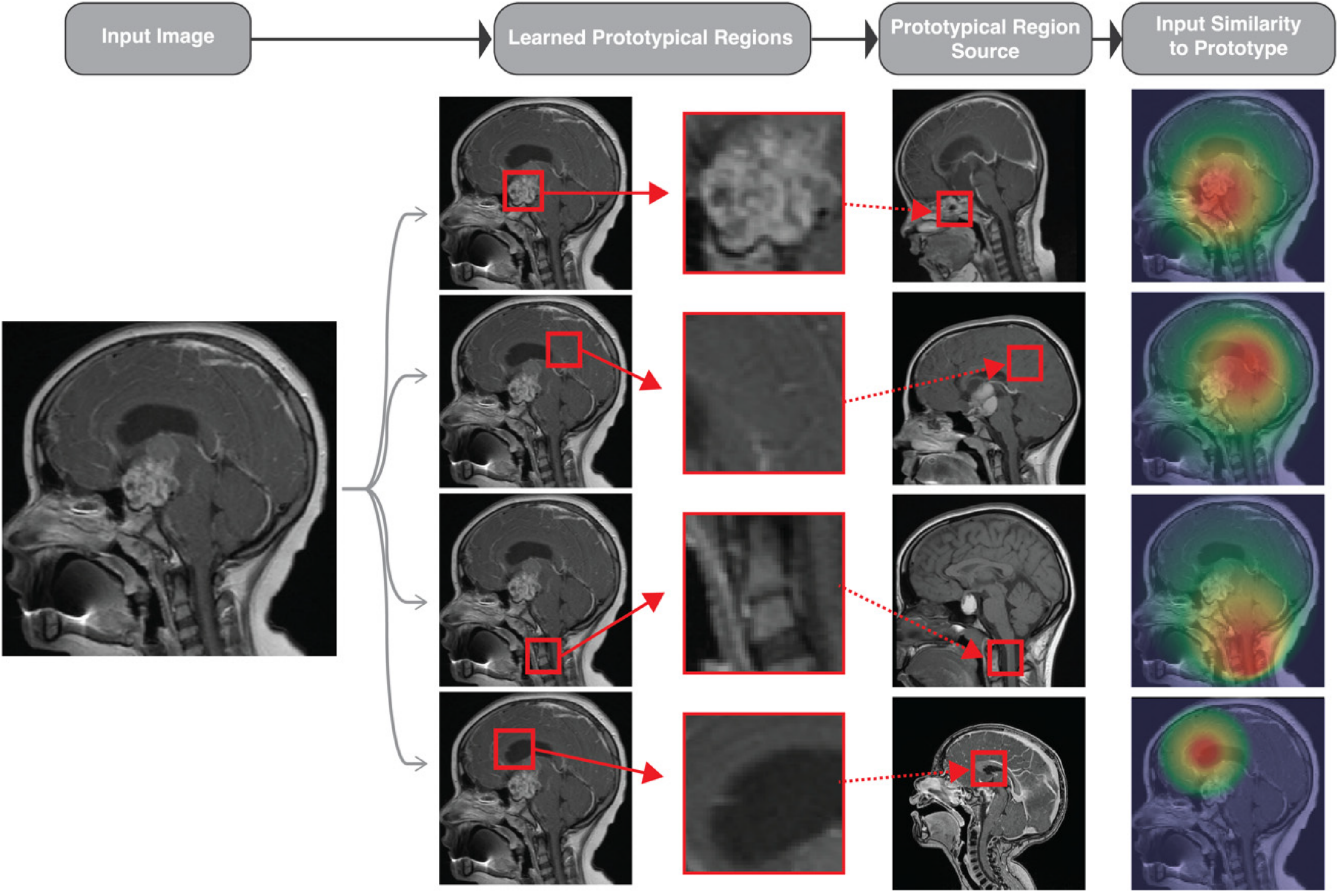
Conceptual rendering of how the “This Looks Like That” (protoPNet) approach could be applied to classify a single sagittal MR image. The input image (left) is a preoperative sagittal contrast-enhanced T1-weighted MR image of a patient diagnosed with a suprasellar tumor.67 The second column shows overlaid bounding boxes generated by a model, highlighting the content like the prototypical parts learned by the algorithm. The third column displays the prototypical parts learned by the model, while the fourth column presents the source images of the prototypical parts. Finally, the rightmost column shows the activation maps indicating the extent to which each prototypical part resembles the test image, with red indicating high and green indicating low resemblance.

The *ante hoc* approach to XAI is significant as it focuses on creating inherently transparent AI systems. Unlike *post hoc* methods that interpret the decisions of trained models, *ante hoc* methods provide transparency from the beginning, making the model's decisions understandable at each stage (4, 28). In the work titled “This Looks Like That,” Chen and colleagues developed the ProtoPNet architecture, which matches inputs to previously learned prototypes (29). This approach could classify a CNS tumor using a single MR image, as shown in Figure 3. The matching facilitates an explanation of what different subregions of a novel input look like when compared to previously seen inputs.

The *ante hoc* approach demands less training data, as the network connections are pre-determined, but it may lead to reduced accuracy due to its inflexibility (30). Nonetheless, the ability to develop specialized AI systems based on existing knowledge can lead to more transparent AI architectures, offering valuable insights and supporting human experts in various tasks.

Several groups have comprehensively reviewed XAI healthcare methods, analyzing technical implementations, challenges, explanation types, scope, interpretability, and the risks of medical interpretability and class activation maps (31, 32). Meeting the user's needs is essential to creating a successful biomedical XAI system. This involves understanding the application context by modeling the biomedical and clinical domains. Key actors must be identified, decision-making processes must be defined, data collection phases must be clarified, and critical elements must be identified, including potential missing data. Combi et al. also stress the importance of measuring, interpreting, and understanding AI systems' usability, usefulness, and interpretability. They propose that explanations are only necessary for XAI models under certain circumstances (32). A domain analysis and user-centered study can determine when explainability is required.

Visual analytics in XAI aim to enhance individuals' comprehension of the data, models, and results generated by AI systems. Even if an AI system's accuracy is not optimal, visual analytics can highlight important areas within an image, enhancing overall performance. However, few visual analytics platforms are tailored to clinical predictive modeling (33–35).

The communication method between the human user and the AI system is a crucial aspect of XAI approaches. Visual analytics approaches use visual representations and interactive interfaces to make data, models, and outputs easily understandable for humans (6, 36, 37). These approaches allow for the rapid analysis of large amounts of data, identifying patterns, and deriving meaningful insights. For example, Villain et al. introduced a visualization technique that can effectively guide a user's attention to specific parts of an image, even when the model's accuracy is relatively low (38). This implies that AI, even with limited accuracy, can be beneficial by enhancing human performance through attention direction. However, it's important to note that this approach can lead to false positives, disrupting workflow by requiring additional time to investigate non-existent issues. This prolongs the work of radiologists, clinicians, and others, potentially harming patients by necessitating unnecessary procedures. Despite these challenges, visual analytics methods specifically designed for clinical settings are still in their infancy (6, 37). Most existing platforms are designed for traditional statistical methods, with only a few catering to predictive modeling. This presents an opportunity to develop AI tools that support clinical decision-making while minimizing risks like those associated with false positives.

## 4 Enhancing the user experience and efficiency of XAI implementation in healthcare with UCD

It is vital to understand the audience, their goals, and the decision-making context to determine the understandability of an explanation (39). Embedding users' needs in AI design principles is crucial to creating efficient and effective XAI clinical decision support systems (CDSS) that meet clinicians' requirements. The computer science community has developed guidelines for AI explanations to achieve this. One such example is the XAI User Needs Library for the medical field (40–42). This library considers various factors related to AI model performance and ethics and was developed in collaboration with engineers, AI experts, and clinical end-users. Design components and a prototype were then created and evaluated based on this library. The results emphasized the importance of managing explanation detail and personalization for the clinical end-user.

Schoonderwoerd et al. developed the DoReMi approach, a user-centered design workflow that considers social contexts for AI-generated explanations in CDSS for diagnosing ADHD (43). This approach allows developers, designers, and clinical end-users to collaborate to create efficient and effective XAI CDSS. Similarly, Cai and colleagues conducted a study using UCD methods to identify user onboarding needs for deploying XAI in prostate cancer diagnosis (44). They found that an AI CDSS should have performance metrics based on human benchmarks, transparent data sources, explicit objectives, and comprehensive information on AI model provenance, tool validation, legal liability, regulatory approval, expected costs, and impacts on workflows, as well as a non-confrontational interface that directs attention instead of correcting the user.

The tool BreastScreening-AI, developed by Calisto et al., aims to customize explainable AI methods for clinical users and their tasks, focusing on the impact on medical workflow, changes in clinicians’ expectations, and the effect on breast cancer classification from mammography imaging (45). The group conducted a follow-up study to assess how different forms of communication enhance the performance of clinical experts when presenting information on their BreastCancer-AI software. The study aimed to evaluate diagnostic time and accuracy, with the experimental variable being the tone of communication for predictions—either assertive or nonassertive (46). The findings indicated that junior clinicians responded better to assertive statements, while junior clinicians showed greater accuracy improvement than their senior counterparts. These studies highlight the importance of understanding the clinical context of AI deployment and the potential benefits of using variable assertiveness to communicate messages with varying confidence levels. For example, the system can use an assertive tone for highly confident predictions and a nonassertive style for predictions with low confidence (<80%) to reflect the level of confidence.

These studies demonstrate the application of UCD in defining design requirements and creating successful XAI prototypes, offering transparency, accountability, and user-friendly tools to improve the user experience and enhance the efficiency and effectiveness of AI implementation.

## 5 Harnessing AI for improved clinical outcomes in neuro-oncology treatment

Extensive testing has been conducted on the feasibility of using AI for clinical treatment of CNS cancers. The current FDA-approved commercial products used for MR imaging in CNS tumor care include AI devices for preoperative neurosurgical and radiation treatment planning. For example, BrainLab AG offers BrainLab Elements and Automatic Registration iMRI for neurosurgical planning and Philips MRCAT Brain for radiation treatment planning. Various automatic segmentation and volumetric analysis software solutions are available, although they are not specifically related to CNS tumors. Here, we focus on preclinical solutions that have not been approved by the FDA but are candidate applications and solutions currently being developed. Table 2 provides further details, and the following sections discuss XAI and UCD efforts in these contexts.

### 5.1 Factors to consider in tailoring UCD guidelines for neuro-oncology

There is an increasing demand for guidelines to help direct the design of XAI systems that support decision-making in clinical neuro-oncology practice (12, 47). Meeting this demand necessitates the inclusion of UCD in the development of XAI (48). Although specific XAI design guidelines for clinical neuro-oncology remain to be defined, some prospective elements have been described.

One of the most directly applicable resources is the INTRPRT framework, a set of UCD guidelines specific to medical imaging AI (12). These guidelines are relevant to neuro-oncology because clinical image analysis and interpretation are the most common AI applications in neuro-oncology (39, 49). Like DoReMi, INTRPRT emphasizes the importance of formative user research, empirical user testing, general assessment of model transparency, and XAI systems for diverse stakeholders. Notably, the authors emphasize that these guidelines are a starting point and must be adapted and refined to the individual context of an AI application to ensure it is tailored to the clinical end-user's needs. This requirement for refinement and adaptation asserts that the successful implementation of XAI in neuro-oncology (or any specific field) merits customized UCD.

From the clinical provider perspective, we can abstract clinical decision-making into two steps: gathering high-quality information and aggregating that information to make decisions (50). For AI to gather high-quality information, the interface between the AI and humans must be designed to ensure that the information is accessible and understandable to the expected clinical user (6, 48, 51). AI can also assist with information aggregation to reduce diagnostic error and cost, improve electronic health record data collection and interpretation, and potentially help make advanced technologies more available in geographic areas without specialized medical facilities (36, 52–61). To leverage this potential, AI technologies must establish trust with clinical decision-makers and demonstrate greater intuitive usability for clinicians (62).

### 5.2 Unlocking the potential of AI-assisted imaging diagnosis in neuro-oncology

Assisting in generating a radiologic differential diagnosis is the most common AI application in neuro-oncology (39, 49). While not currently available, a potential outcome of AI-assisted imaging diagnosis would be to no longer depend upon invasive tissue biopsy to obtain the biological data needed to guide therapy. Using pediatric Adamantinomatous Craniopharyngioma (ACP) as an example, the current diagnosis includes neurosurgical biopsy more than 91% of the time (63). However, radiotherapy alone could be an effective treatment for ACP without any neurosurgical intervention (64, 65). Neuroradiologists can identify ACP from preoperative MRI with an average accuracy of 86%. Existing deep learning algorithms perform equally as well at this task (66, 67). While AI performance can assist neuroradiologists in developing an efficient and thorough differential diagnosis, it does not provide enough evidence to eliminate the need for neurosurgical biopsy in diagnosis mitigation. Another practical example of XAI in neuro-oncology is AutoRAPNO, a completely automated system for segmenting pediatric medulloblastomas, high-grade gliomas, and tumors that have seeded the leptomeninges, based on the Response Assessment in Pediatric Neuro-Oncology (RAPNO) guidelines (68). Integrating AI support with guidelines like RAPNO has yielded a more reproducible and standardized assessment of treatment response across clinicians, specifically in patients with low-grade glioma (69). By efficiently and consistently applying consensus recommendations, such as those defined by RANO (70) or RAPNO working groups (71–73), XAI tools could facilitate clinical decision-making and be used for continuous guideline improvement.

It is essential to consider workflow aspects related to clinical AI deployment. Chakrabarty et al. developed Integrative Imaging Informatics for Cancer Research: Workflow Automation for Neuro-oncology (I3CR-WANO). This AI-driven framework transforms raw MRI DICOM data of patients with high- and low-grade gliomas into quantitative tumor measurements (74). This work can streamline clinical workflows, support clinical decision-making by automating tumor segmentation and characterization, and help curate large-scale neuro-oncology data sets. Finally, Zeineldin et al. presented a new explainability framework, NeuroXAI, to assist in interpreting the behavior of DL networks using state-of-the-art visualization attention maps (75). NeuroXAI is *post hoc*, applicable to any deep neural models, providing insights into their behavior. These examples highlight the significance of XAI methods in medical image analysis. NeuroXAI aids CNN analysis by supplying individual activation maps for each internal layer, helping guide expert users. However, the vague information from attention-based XAI can cause information overload and alert fatigue, leading to wasted time procedures (31).

AI in MR interpretation also includes radiomics and radiogenomics, which analyze detailed data from medical imaging to predict diagnosis, molecular stratification, and personalized treatment trials (76). These AI outputs can be used in various applications, including hypothesizing, predicting patient response to radiotherapy, identifying radiation necrosis, virtual biopsy, predicting tumor mutational status, and classifying brain tumor images. These progressions are of particular significance in the medical management of brain metastases, an abundant terminal illness (77). By providing *in vivo* markers of molecular and spatial heterogeneity, AI-based radiomic and radiogenomic methods can support the division of patients into improved initial diagnostic and therapeutical pathways and for dynamic treatment monitoring (78). Furthermore, integrating image analysis, deep learning, and radiomics can help enhance radiotherapy programs (79). Technological advances in areas like radiomics can revamp the standard of care for individuals diagnosed with brain metastases, providing personalized medical attention and treatment.

### 5.3 Enhancing patient outcomes through AI in neurosurgery

AI applications are rapidly adopted in neurosurgery, offering a variety of clinical advantages such as enhanced lesion characterization, predictions of surgical outcomes and potential complications, and projecting healthcare costs (80). AI has made a significant impact in preoperative neurosurgical planning and is expected to continue to do so to enhance the care and outcomes of patients in this area. During pre-operative stages, AI has been employed to assess overall health, select the appropriate operative approach, and inform patients of the risks involved in the procedure. Intraoperatively, AI is commonly used to aid with vital sign monitoring. For example, radiomics and AI are used to forecast intraoperative changes in visual evoked potentials or cerebral spinal fluid leaks, as well as the estimated total expense (81, 82). Surgical planning tools have largely been built on population-scale data but have yet to be implemented on an individual patient level. For many patients with a CNS tumor, there is an opportunity for improved AI solutions to contribute to personalized decision-making around calculating surgical risk and operative approach (83). Furthermore, there is increasing interest in using AI in neurosurgical training, and further research is needed (84). AI enhances neurosurgical outcomes, and incorporating user-centered design ensures these advancements meet the specific needs of patients and healthcare providers.

### 5.4 Using AI to optimize radiotherapy for CNS tumor treatment

Radiation therapy is an essential tool for treating cancer. AI-based studies have investigated five areas of radiotherapy: image reconstruction, image registration, image segmentation, image synthesis, and automatic treatment planning (85). An example of the application of AI in clinical radiotherapy includes a parameter optimization system that automates portions of dose distribution without interfering with physicians inputting clinically viable constraints (86). In another example, deep reinforcement learning was used to design customized treatment regimens for glioblastoma patients, leveraging a proliferation invasion model to simulate tumor growth and its response to therapy (87). The AI framework was evaluated for designing chemo-radiation therapy regimens based on patient characteristics to construct individualized treatments better suited to each patient's needs than the conventional regimens recommended by clinical trials. Notably, the authors emphasize that this framework is designed to be completely safe *because* it merely provides decision support to clinicians while still allowing clinicians to make the final call on whether to implement or discontinue the therapy. When clinicians maintain agency over the decision-making process, it does provide some safety to patients; it is crucial to continuously verify that the interactions between clinicians and AI remain consistent concerning patient outcomes. The implementation of high-performance computing, artificial intelligence, and advances in imaging technology have revolutionized the role of imaging in medicine (88).

### 5.5 Interface system development options for XAI in neuro-oncology

A variety of resources exist to support the implementation of XAI in neuro-oncology. Local resources, public databases, and Federated databases, which have been gaining popularity, can all be used as data sources for these XAI models. Notable resources within neuro-oncology include The Cancer Imaging Archive and the Pediatric Brain Tumor Atlas; others are listed in Table 3. Developers use AI-specific languages and frameworks to train models on high-performance architecture like GPU servers. Interface system development options include Python or R packages, full-stack web development, and software libraries, allowing for customization and scalability of medical imaging and web analytics. It's crucial to consider the distinctions between training and production models and the availability of explainability methods.

**Table 3:** Available data resources for developing XAI models for CNS tumors. IDs reflect entries in Figure 1.

ID	Name	Description	Controlled	URL
DR1	Figshare brain tumor dataset	This brain tumor dataset consists of 3064 T1-weighted contrast-enhanced images from 233 patients, with three types of brain tumors: meningioma (708 slices), glioma (1426 slices), and pituitary tumor (930 slices). The dataset is split into four subsets, with each subset containing 766 slices, and includes patient information and tumor border information to generate tumor masks.	N	https://figshare.com/articles/dataset/brain_tumor_dataset/1512427
DR2	Kaggle brain tumor MRI dataset	This dataset consists of 7023 human brain MRI images classified into four classes: glioma, meningioma, no tumor, and pituitary	N	https://www.kaggle.com/datasets/masoudnickparvar/brain-tumor-mri-dataset
DR3	TCIA—ReMIND	The Brain Resection Multimodal Imaging Database (ReMIND) contains pre- and intra-operative imaging data, including preoperative MRI, intraoperative ultrasound, and intraoperative MRI, from 114 patients who underwent image-guided tumor resection between 2018 and 2022, along with segmentations of various structures, and this dataset aims to support computational research and neurosurgical training in brain shift analysis and image interpretation	N	https://wiki.cancerimagingarchive.net/pages/viewpage.action?pageId=157288106
DR4	Pediatric Brain Tumor Atlas	The Pediatric Brain Tumor Atlas (PBTA) is a collaborative project that aims to accelerate discoveries for treating brain tumors in children by providing comprehensive datasets including genomic, clinical, imaging, and histology data, which can be accessed through the Gabriella Miller Kids First Data Resource Portal and analyzed using the Cavatica platform, with funding support from over 50 foundations.	Y	https://cbtn.org/pediatric-brain-tumor-atlas
DR5	Br35H—Brain Tumor Detection 2020	This dataset includes 3000 axial T1-weighted MRI images (in jpeg format) annotated as either tumorous or nontumorous	N	https://www.kaggle.com/datasets/ahmedhamada0/brain-tumor-detection
DR6	CBTN-CONNECT-DIPGr-ASNR-MICCAI BraTS-PEDs 2023	This is a large dataset of annotated high-grade glioma in children, using manually annotated MRI scans to evaluate predicted tumor segmentations generated by algorithms submitted to the CBTN-CONNECT-DIPGr-ASNR-MICCAI BraTS-PEDs 2023 Challenge.	Y	https://www.synapse.org/#!Synapse:syn51156910/wiki/622461
DR7	BraTS 2020—Brain Tumor Segmentation	The BraTS multimodal scans consist of native, post-contrast T1-weighted, T2-weighted, and T2 Fluid Attenuated Inversion Recovery (T2-FLAIR) volumes, which were obtained from multiple institutions using different protocols and scanners, and are available as NIfTI files. These scans were manually segmented by one to four raters following an approved annotation protocol by experienced neuro-radiologists, including the GD-enhancing tumor (ET), peritumoral edema (ED), and the necrotic and non-enhancing tumor core (NCR/NET) labels, and have undergone pre-processing to ensure consistency in spatial resolution (1 mm^3^) and skull-stripping. Note: this is one example of many BraTS datasets that are available	N	https://www.kaggle.com/datasets/awsaf49/brats2020-training-data

### 5.6 Measuring usability and benefits with UCD tools

To ensure scientific rigor, UCD is recommended to follow an explicit hypothesis testing approach. This involves specifying the hypothesis and testing methods. Table 4 provides a selection of UCD tools that can be used to measure the usability and benefit of software solutions. For those looking for a comprehensive toolkit of explainable artificial intelligence (XAI) methods, UC Berkeley's website *uxai.design* is a great resource. This website has extensive information on defining user groups, including decision-makers, affected users or patients, regulatory bodies, and internal stakeholders. The toolkit provides XAI methods thoughtfully categorized into “what,” “why,” “why not,” “what if,” “how,” “how to be,” “how to be still,” “how confident,” “what data,” “what outputs,” and “how it works” to help facilitate design decisions. These methods are further discussed in the context of onboarding, regular interaction, system errors, and system updates. Additionally, the website discusses methods like *ante hoc* or *post hoc* approaches and global or local explanations. Evaluation guidance includes functional, operational, usability, and safety requirements. Finally, validation is conducted using the Dolshi-Velasquez structure, with functionally grounded, human, and application-level evaluation.

**Table 4:** Evaluation methods related to UCD of clinical XAI systems.

Name	Description	Approach	Abstraction level	Evaluation level
System usability score (SUS)	The SUS measures the perceived usability of a system through a 10-statement questionnaire. Participants rate their agreement on a five-point scale, with scores converted to a standardized score ranging from 0 to 100. SUS is widely used in industry and academia to evaluate software and websites	Survey	Human grounded	Usability
NASA task load index (TLX)	The TLX measures mental workload across six dimensions, including demand, performance, effort, and frustration. It helps researchers understand how tasks affect cognitive and physical demands and improve system design and human performance	Survey	Human grounded	Workload
ICE-T	The ICE-T equation is a value equation that includes four components: T, I, E, and C. “T” represents a visualization's capacity to answer a broad range of questions about data in a minimal amount of time. “I” pertains to a visualization's ability to stimulate insights or thought-provoking inquiries about data. “E” refers to a visualization's essence, its capacity to convey an overall impression or summary of the data. *“*C*”* represents a visualization's ability to instill confidence, knowledge, and trust about the data, domain, and context	Survey	Human grounded	Comprehension
Model performance	Different metrics are used to evaluate an AI model's performance depending on the task and objective. Accuracy is commonly used for classification tasks, while precision and recall are used for imbalanced classes. The F1 score is used to balance precision and recall. For regression tasks, mean squared error and mean absolute error are used, with root mean squared error providing a more interpretable value. AUC is used in binary classification and mAP in object detection. Cross-validation can give a more reliable estimate of performance. The choice of metric depends on the specific task and requirements	Quantitative	Application grounded	Performance

## 6 Discussion and conclusion

XAI tools are becoming increasingly important in clinical settings, but their reliable implementation is hindered by mistrust and other challenges, such as usability, validity, and utility. Co-creating AI technology with clinicians can increase trust and help meet their expectations. A user-centered design approach can target existing and newer methods of AI technology for improved implementation.

### 6.1 Multi-disciplinary skills are essential for designing and developing successful clinical neuro-oncology AI systems

The goal of utilizing UCD to steer clinical AI development is for computer scientists, engineers, and medical experts to collaboratively create solutions that directly meet the needs of medical experts. By formalizing and following a UCD process, we can include visual analytics engineers, user experience designers, and computer scientists in the discussion, emphasizing artistic creativity backed by scientifically rigorous functionality. Previously, AI development was primarily performed by computer scientists with limited or no clinical expertise. Designing a successful clinical AI system necessitates a multidisciplinary team skilled in various areas, including clinical medicine, computer science, software engineering, artificial intelligence, design, observational studies, and other mixed methods. This team must work together to tackle the technical and humanistic challenges of the endeavor, such as devising a solution tailored to the target clinical user base and understanding how to set expectations for the system's function and benefits. The complex computational environments needed to handle medical data, build interactive AI systems, and deploy and evaluate these systems hinder the development and standardization of UCD clinical AI systems. As such, a wise starting point for the multi-disciplinary research community would be to formally define a flexible software ecosystem to serve as the foundation for development.

### 6.2 User-centered design can help mitigate AI-induced biases in clinical decision-making, resulting in systems that improve patient outcomes and clinical workloads

Implementing clinical AI for MRI interpretation in neuro-oncology patient management requires standardized imaging protocols, data sharing for generalizability, and federated learning. Standardization ensures consistency and accuracy in images acquired across different settings. Generalizability allows for inferences for a large population by studying a smaller sample, while federated learning enables collaboration on model training without sharing sensitive patient data.

Clinical AI applications have the potential to introduce bias into medical decisions, impacting accuracy, fairness, and patient safety. Bias stemming from data, algorithmic, and human factors can lead to skewed AI results. It is important to consistently review and update datasets to mitigate bias and uncertainty in AI and ensure diversity, representativeness, and transparency. Moreover, it is crucial to critically examine AI to prevent the perpetuation of existing inequalities and to offer clear explanations for AI decisions accessible by clinicians, thus mitigating concerns about bias. However, this is only part of the solution.

Emerging research indicates that human experts variably incorporate AI information into their decision-making, sometimes over- or underweighting predictive information, deviating from normal decision-making processes (16). This human behavior highlights the need for UCD to create clinical AI tools (12, 89). Through UCD, we can define, anticipate, and mitigate these deviations, ensuring clinical AI solutions are seamlessly embedded in existing workflows and meet stakeholder expectations.

To effectively mitigate AI-induced biases, using robust quantitative metrics, it is essential to assess the performance and suitability of XAI systems in clinical decision-making. Key metrics include accuracy and precision, which measure the correctness of the model's predictions. Fidelity assesses how explanations reflect the model's decision-making process, while comprehensibility quantifies the ease with which clinicians understand these explanations. Trust and satisfaction scores, gathered through clinician surveys, gauge the system's acceptance and usability. Time efficiency measures the time required for clinicians to interpret explanations and make decisions, ensuring practicality in fast-paced environments. Using tools like confusion matrices and Receiver Operating Characteristic curves, error analysis evaluates error types and frequencies, providing insights into the model's reliability.

In addition to these metrics, leveraging established metrics from Human-Computer Interaction (HCI) and social sciences can further enhance the evaluation of XAI in clinical settings. HCI metrics, such as time, error rates, and comprehension, are essential for evaluating the efficiency and effectiveness of XAI systems. Social science metrics, including communication and trust, are crucial for understanding the interaction between clinicians and AI systems. While some metrics, such as accuracy and error rates, are straightforward to collect, others, like trust and communication, may require more sophisticated methods, including surveys and observational studies, to gather meaningful quantitative data.

These metrics collectively provide a comprehensive framework for evaluating XAI systems, ensuring they meet high clinical standards. However, more work is needed to expand these quantitative metrics to develop even more comprehensive XAI systems in the future.

### 6.3 User-centered XAI tools aim to improve clinicians' performance and fundamentally differ from AI solutions that aim to complete automation and replace humans in decision-making

Contrary to the prevailing notion in many studies, we believe AI is not meant to replace human experts in radiology but to enhance their capabilities. AI should empower experts to make more informed, efficient, and accurate decisions. Guided by UCD, AI applications can be tailored to specific clinical decisions, improving patient management. It is critical to fully assess the risks associated with AI errors within the human expert's workflow.

Generative AI systems are being studied for their benefits in ambient audio and video recording within clinical settings, enhancing record-keeping and shared decision-making and theoretically improving efficiency in neuro-oncology care. They are also being explored to expedite MRI acquisition and generate realistic MR scans for neuro-oncology patients who struggle to remain still. However, generative AI is prone to hallucinations, where the AI generates incorrect or misleading information that is difficult or impossible for humans to perceive. These hallucinations can lead to inaccurate records, misinformed clinical decisions, increased liability, flawed images, incorrect diagnoses, repeat scans, increased clinician workload, patient stress, and overall healthcare costs.

In visual analytics, generative AI is being studied to create personalized user interfaces that help users efficiently distill large volumes of data. Although functional, hallucinations at this stage could decrease clinical productivity and hinder expert adoption. Generative AI can also generate radiology impressions, potentially increasing efficiency in patient management. However, AI-generated text with random measurement values can lead to incorrect treatments and confound clinical trials. Synthetic MR models are being explored to speed up imaging, preserve patient privacy, and predict patient treatment response. Still, hallucinations in these images can pose significant risks for accurate diagnosis and treatment planning.

Each example carries a unique risk profile, but none are excluded from real-world applications. AI tools developed with a UCD focus improve human performance through AI support rather than replacing humans and concentrating solely on AI performance. Following UCD principles allows us to define and weigh these risks among stakeholders, setting expectations and leading to a more efficient allocation of resources and capital to create AI systems that can swiftly and effectively impact patient management.

### 6.4 Designing XAI systems for clinical decision-making involves navigating the trade-off between model accuracy and transparency

Accuracy and transparency are crucial for ensuring patient safety and clinician trust in high-risk environments. To define clinical XAI requirements, it is essential to consider the specific needs and constraints of the clinical environment, including understanding clinical workflows, decision types, and the impact on patient outcomes. Increasing model accuracy often reduces transparency, as complex models like deep neural networks are less interpretable than simpler models such as decision trees. For critical decisions, where transparency is paramount, simpler models can build trust and ensure clinicians understand the decision-making process. For less critical decisions, more complex models can maximize performance. Hybrid models, which combine interpretable models with high-performance models, can balance transparency and accuracy by leveraging both strengths. *Post-hoc* explanation techniques, such as LIME and SHAP, provide insights into complex models without altering their structure, maintaining high performance while offering transparency.

To effectively implement these strategies, it is imperative to conduct comprehensive user research to understand clinicians' needs and preferences regarding explanations. Additionally, we can utilize the existing clinical decision-support literature to identify and further enhance best practices for evaluating clinical XAI tools (90). Developing a modular explanation system offering high-level summaries with detailed drill-down options can help balance transparency and performance. Continuous learning and adaptation mechanisms allow the system to evolve based on new data and user interactions, maintaining a balance between transparency and performance. Ensuring regulatory compliance and usability is essential, as the system must meet healthcare regulations and integrate seamlessly into clinical workflows. By employing these strategies, it is possible to design XAI systems that effectively balance accuracy and transparency, ensuring they are both practical and trustworthy in clinical decision-making. This approach provides a comprehensive framework for developing XAI systems that meet the high standards required in clinical environments, fostering trust and reliability among users.

### 6.5 Establishing a standardized UCD process for designing and evaluating AI-based healthcare technologies across different contexts will help to inform effective and safe solutions

UCD emphasizes that the development, design, and evaluation process is as important as the resulting software. This importance is warranted because clinical settings are variable depending on the country and general accessibility to healthcare resources. Due to this observation and general regulatory trends, we do not foresee a generalizable AI solution being implemented across all settings; instead, we envision a landscape in which AI functionality will need to be packaged for each clinical application. Therefore, we must adopt a rigorous scientific approach in our UCD efforts to derive reusable clinical AI design templates. In addition to providing a means for quick and reliable deployment of clinical AI tools, such a process would also enable a method to evaluate clinical AI scientifically at a global level. Understanding this process is crucial in developing effective technologies that improve medical outcomes and patient safety.

Guidelines like INTRPRT, designed to enhance transparency in AI and machine learning systems, must be regularly updated to remain relevant amidst rapid technological advancements. Establishing a schedule for periodic reviews and updates is crucial. This can be facilitated by a dedicated committee comprising clinicians, AI researchers, ethicists, and regulatory experts. Community involvement through workshops, conferences, and online forums ensures that the guidelines reflect the latest developments and best practices. Developing adaptive frameworks allows for flexible and targeted updates, ensuring the guidelines can quickly incorporate new techniques and findings. Continuous education and training programs keep clinicians and developers informed about the latest advancements and updates, fostering a culture of continuous learning. Leveraging automated tools to monitor research publications, regulatory changes, and technological advancements can provide timely alerts for necessary updates. Establishing a feedback loop with end-users, such as clinicians and developers, ensures that the guidelines remain practical and applicable in real-world settings. These strategies collectively ensure that guidelines like INTRPRT stay current and effective, supporting transparency and best practices in clinical decision-making.

In conclusion, XAI tools are available to support more accurate and efficient clinical decision-making, especially in neuro-oncology. UCD principles can ensure user trust and reduce bias and discrepancies, resulting in a flexible software ecosystem tailored to clinical end-user needs. Ultimately, UCD has the promise to increase the translational efficacy of clinical AI solutions to support clinicians and improve patient care.

## References

[1] Khalighi S Reddy K Midya A Pandav KB Madabhushi A Abedalthagafi M. Artificial intelligence in neuro-oncology: advances and challenges in brain tumor diagnosis, prognosis, and precision treatment. NPJ Precis Oncol. (2024) 8(1):1–12. 10.1038/s41698-024-00575-0 38553633 PMC10980741

[2] Abdel Razek AAK Alksas A Shehata M AbdelKhalek A Abdel Baky K El-Baz A Clinical applications of artificial intelligence and radiomics in neuro-oncology imaging. Insights Imaging. (2021) 12(1):152. 10.1186/s13244-021-01102-6 34676470 PMC8531173

[3] Arrieta AB Díaz-Rodríguez N Del Ser J Bennetot A Tabik S Barbado A Explainable artificial intelligence (XAI): concepts, taxonomies, opportunities and challenges toward responsible AI. Inf Fusion. (2020) 58:82–115. 10.1016/j.inffus.2019.12.012

[4] Minh D Wang HX Li YF Nguyen TN. Explainable artificial intelligence: a comprehensive review. Artif Intell Rev. (2022) 55(5):3503–68. 10.1007/s10462-021-10088-y

[5] Gerlings J Jensen MS Shollo A. Explainable AI, but explainable to whom? An exploratory case study of xAI in healthcare. In: Handbook of Artificial Intelligence in Healthcare: Vol 2: Practicalities and Prospects. Berlin: Springer Nature (2022). p. 169–98.

[6] Ooge J Stiglic G Verbert K. Explaining artificial intelligence with visual analytics in healthcare. WIRES Data Min Knowl Discov. (2022) 12(1):e1427. 10.1002/widm.1427

[7] Antoniadi AM Du Y Guendouz Y Wei L Mazo C Becker BA Current challenges and future opportunities for XAI in machine learning-based clinical decision support systems: a systematic review. Appl Sci. (2021) 11(11):5088. 10.3390/app11115088

[8] Mohamed Musthafa MM Mahesh TR Vinoth Kumar V Guluwadi S. Enhancing brain tumor detection in MRI images through explainable AI using grad-CAM with resnet 50. BMC Med Imaging. (2024) 24(1):107. 10.1186/s12880-024-01292-7 38734629 PMC11088067

[9] Jekel L Brim WR von Reppert M Staib L Cassinelli Petersen G Merkaj S Machine learning applications for differentiation of glioma from brain metastasis—a systematic review. Cancers (Basel). (2022) 14(6):1369. 10.3390/cancers14061369 35326526 PMC8946855

[10] Wang S Kim SJ Poptani H Woo JH Mohan S Jin R Diagnostic utility of diffusion tensor imaging in differentiating glioblastomas from brain metastases. Am J Neuroradiol. (2014) 35(5):928–34. 10.3174/ajnr.A3871 24503556 PMC7964538

[11] Agarwal N Moehring A Rajpurkar P Salz T. Combining Human Expertise with Artificial Intelligence: Experimental Evidence from Radiology (No. w31422). Cambridge, MA: National Bureau of Economic Research (2023). p. 1–126.

[12] Chen H Gomez C Huang CM Unberath M. Explainable medical imaging AI needs human-centered design: guidelines and evidence from a systematic review. NPJ Digit Med. (2022) 5(1):1–15. 10.1038/s41746-022-00699-2 36261476 PMC9581990

[13] Voigtlaender S Pawelczyk J Geiger M Vaios EJ Karschnia P Cudkowicz M Artificial intelligence in neurology: opportunities, challenges, and policy implications. J Neurol. (2024):2258–73. 10.1007/s00415-024-12220-8 38367046 PMC12239762

[14] Familiar AM Fathi Kazerooni A Vossough A Ware JB Bagheri S Khalili N Towards consistency in pediatric brain tumor measurements: challenges, solutions, and the role of AI-based segmentation. Neuro-Oncol. (2024):1557. 10.1093/neuonc/noae093 38769022 PMC11376457

[15] Eltawil FA Atalla M Boulos E Amirabadi A Tyrrell PN. Analyzing barriers and enablers for the acceptance of artificial intelligence innovations into radiology practice: a scoping review. Tomogr Ann Arbor Mich. (2023) 9(4):1443–55. 10.3390/tomography9040115 PMC10459931 37624108

[16] Yu F Moehring A Banerjee O Salz T Agarwal N Rajpurkar P. Heterogeneity and predictors of the effects of AI assistance on radiologists. Nat Med. (2024) 30(3):837–49. 10.1038/s41591-024-02850-w 38504016 PMC10957478

[17] Chan DC Gentzkow M Yu C. Selection with variation in diagnostic skill: evidence from radiologists*. Q J Econ. (2022) 137(2):729–83. 10.1093/qje/qjab048 35422677 PMC8992547

[18] Kwong JC Nguyen DD Khondker A Kim JK Johnson AE McCradden MM When the model trains you: induced belief revision and its implications on artificial intelligence research and patient—a case study on predicting obstructive hydronephrosis in children. NEJM AI. (2024) 1(2):AIcs2300004. 10.1056/AIcs2300004

[19] Lewrick M Link P Leifer L. The Design Thinking Toolbox: A Guide to Mastering the Most Popular and Valuable Innovation Methods. Hoboken, NJ: John Wiley & Sons (2020). p. 1–320.

[20] Munzner T. A nested model for visualization design and validation. IEEE Trans Vis Comput Graph. (2009) 15(6):921–8. 10.1109/TVCG.2009.111 19834155

[21] Wang Q Huang K Chandak P Zitnik M Gehlenborg N. Extending the nested model for user-centric XAI: a design study on GNN-based drug repurposing. IEEE Trans Vis Comput Graph. (2023) 29(1):1266–76. 10.1109/TVCG.2022.3209435 36223348

[22] Salahuddin Z Woodruff HC Chatterjee A Lambin P. Transparency of deep neural networks for medical image analysis: a review of interpretability methods. Comput Biol Med. (2022) 140:105111. 10.1016/j.compbiomed.2021.105111 34891095

[23] Ivanovs M Kadikis R Ozols K. Perturbation-based methods for explaining deep neural networks: a survey. Pattern Recognit Lett. (2021) 150:228–34. 10.1016/j.patrec.2021.06.030

[24] Lundberg SM Lee SI. A unified approach to interpreting model predictions. In: Guyon I, Von Luxburg U, Bengio S, Wallach H, Fergus R, Vishwanathan S, et al., editors. Advances in Neural Information Processing Systems. Vol 30. Long Beach, CA: Curran Associates, Inc. (2017). Available online at: https://proceedings.neurips.cc//paper/2017/hash/8a20a8621978632d76c43dfd28b67767-Abstract.html (accessed August 17, 2021).

[25] Ribeiro MT Singh S Guestrin C. “Why Should I Trust You?”: Explaining the Predictions of Any Classifier. ArXiv160204938 Cs Stat. Cornell University (2016). Available online at: http://arxiv.org/abs/1602.04938 (accessed March 2, 2022).

[26] Hailemariam Y Yazdinejad A Parizi RM Srivastava G Dehghantanha A. An empirical evaluation of AI deep explainable tools. In: 2020 IEEE Globecom Workshops (GC Wkshps); Taipei, Taiwan. Institute for Electrical Engineers. 2020:1–6. 10.1109/GCWkshps50303.2020.9367541

[27] Jin W Li X Hamarneh G. Evaluating explainable AI on a multi-modal medical imaging task: can existing algorithms fulfill clinical requirements? Proc AAAI Conf Artif Intell. (2022) 36(11):11945–53. 10.1609/aaai.v36i11.21452

[28] Quinn TP Jacobs S Senadeera M Le V Coghlan S. The three ghosts of medical AI: can the black-box present deliver? Artif Intell Med. (2022) 124:102158. 10.1016/j.artmed.2021.102158 34511267

[29] Chen C Li O Tao D Barnett A Rudin C Su JK. This looks like that: deep learning for interpretable image recognition. In: Wallach H, Larochelle H, Beygelzimer A, d'Alché-Buc F, Fox E, Garnett R, editors. Advances in Neural Information Processing Systems. Vol 32. Vancouver, BC: Curran Associates, Inc. (2019). Available online at: https://proceedings.neurips.cc/paper/2019/hash/adf7ee2dcf142b0e11888e72b43fcb75-Abstract.html (accessed December 21, 2022).

[30] Greene CS Costello JC. Biologically informed neural networks predict drug responses. Cancer Cell. (2020) 38(5):613–5. 10.1016/j.ccell.2020.10.014 33096022

[31] Bharati S Mondal MRH Podder P. A review on explainable artificial intelligence for healthcare: why, how, and when? IEEE Trans Artif Intell. (2024):1429–1442. 10.1109/TAI.2023.3266418

[32] Combi C Amico B Bellazzi R Holzinger A Moore JH Zitnik M A manifesto on explainability for artificial intelligence in medicine. Artif Intell Med. (2022) 133:102423. 10.1016/j.artmed.2022.102423 36328669

[33] Severn C Suresh K Görg C Choi YS Jain R Ghosh D. A pipeline for the implementation and visualization of explainable machine learning for medical imaging using radiomics features. Sensors. (2022) 22(14):5205. 10.3390/s22145205 35890885 PMC9318445

[34] Mörth E Haldorsen IS Bruckner S and Smit NN. Paraglyder: probe-driven interactive visual analysis for multiparametric medical imaging data. In: Magnenat-Thalmann N Stephanidis C Wu E editors. Advances in Computer Graphics. *Lecture Notes in Computer Science*. Springer International Publishing (2020):351–63. 10.1007/978-3-030-61864-3_29

[35] Grist JT Withey S MacPherson L Oates A Powell S Novak J Distinguishing between paediatric brain tumour types using multi-parametric magnetic resonance imaging and machine learning: a multi-site study. NeuroImage Clin. (2020) 25:102172. 10.1016/j.nicl.2020.102172 32032817 PMC7005468

[36] Faiola A Srinivas P Duke J. Supporting clinical cognition: a human-centered approach to a novel ICU information visualization dashboard. AMIA Annu Symp Proc AMIA Symp. (2015) 2015:560–9. 26958190 PMC4765655

[37] Alicioglu G Sun B. A survey of visual analytics for explainable artificial intelligence methods. Comput Graph. (2022):502–20. 10.1016/j.cag.2021.09.002

[38] Villain E Mattia GM Nemmi F Péran P Franceries X Le Lann MV. Visual interpretation of CNN decision-making process using simulated brain MRI. 2021 IEEE 34th International Symposium on Computer-Based Medical Systems (CBMS) (2021). p. 515–20. 10.1109/CBMS52027.2021.00102

[39] Kann BH Hosny A Aerts HJWL. Artificial intelligence for clinical oncology. Cancer Cell. (2021) 39(7):916–27. 10.1016/j.ccell.2021.04.002 33930310 PMC8282694

[40] He X Hong Y Zheng X Zhang Y. What are the users’ needs? Design of a user-centered explainable artificial intelligence diagnostic system. Int J Hum Comput Interact. (2023) 39(0):1519–42. 10.1080/10447318.2022.2095093

[41] Leslie D. Understanding artificial intelligence ethics and safety. arXiv [preprint] arXiv:1906.05684. (2019):1–97.

[42] Sokol K Flach P. Explainability fact sheets: a framework for systematic assessment of explainable approaches. In: Proceedings of the 2020 Conference on Fairness, Accountability, and Transparency. ACM (2020):56–67. 10.1145/3351095.3372870

[43] Schoonderwoerd TAJ Jorritsma W Neerincx MA van den Bosch K. Human-centered XAI: developing design patterns for explanations of clinical decision support systems. Int J Hum-Comput Stud. (2021) 154:102684. 10.1016/j.ijhcs.2021.102684

[44] Cai CJ Winter S Steiner D Wilcox L Terry M. “Hello AI”: uncovering the onboarding needs of medical practitioners for human-AI collaborative decision-making. Proc ACM Hum-Comput Interact. (2019) 3(3 CSCW):1. 10.1145/335920634322658

[45] Calisto FM Santiago C Nunes N Nascimento JC. BreastScreening-AI: evaluating medical intelligent agents for human-AI interactions. Artif Intell Med. (2022) 127:102285. 10.1016/j.artmed.2022.102285 35430044

[46] Calisto FM Fernandes J Morais M Santiago C Abrantes JM Nunes N Assertiveness-based agent communication for a personalized medicine on medical imaging diagnosis. In: Proceedings of the 2023 CHI Conference on Human Factors in Computing Systems. ACM (2023):1–20. 10.1145/3544548.3580682

[47] Kotecha R Aneja S. Opportunities for integration of artificial intelligence into stereotactic radiosurgery practice. Neuro-Oncol. (2021) 23(10):1629–30. 10.1093/neuonc/noab169 34244803 PMC8485447

[48] Triberti S Durosini I La Torre D Sebri V Savioni L Pravettoni G. Artificial intelligence in healthcare practice: how to tackle the “human” challenge. In: Lim CP Chen YW Vaidya A Mahorkar C Jain LC, editors. Handbook of Artificial Intelligence in Healthcare: Vol 2: Practicalities and Prospects. Intelligent Systems Reference Library. Springer International Publishing; 2022:43–60. 10.1007/978-3-030-83620-7_2

[49] Fischer C Petriccione M Donzelli M Pottenger E. Improving care in pediatric neuro-oncology patients: an overview of the unique needs of children with brain tumors. J Child Neurol. (2016) 31(4):488–505. 10.1177/0883073815597756 26245798 PMC5032907

[50] Akbari Z Unland R. A powerful holonic and multi-agent-based front-End for medical diagnostics systems. In: Lim CP Vaidya A Jain K Mahorkar VU Jain LC, editors. Handbook of Artificial Intelligence in Healthcare: Vol. 1—Advances and Applications. Intelligent Systems Reference Library. Springer International Publishing (2022):313–52. 10.1007/978-3-030-79161-2_13

[51] Explainable AI. But explainable to whom? In: Lim C-P, Chen Y-W, Vaidya A, Mahorkar C, Jain LC, editors. An Exploratory Case Study of XAI in Healthcare. SpringerLink. (2022). p. 169–98. Available online at: https://link.springer.com/chapter/10.1007/978-3-030-83620-7_7 (accessed November 20, 2022).

[52] Acampora G Cook DJ Rashidi P Vasilakos AV. A survey on ambient intelligence in healthcare. Proc IEEE. (2013) 101(12):2470–94. 10.1109/JPROC.2013.2262913 PMC3890262 24431472

[53] Fazal MI Patel ME Tye J Gupta Y. The past, present and future role of artificial intelligence in imaging. Eur J Radiol. (2018) 105:246–50. 10.1016/j.ejrad.2018.06.020 30017288

[54] Rauschecker AM Rudie JD Xie L Wang J Duong MT Botzolakis EJ Artificial intelligence system approaching neuroradiologist-level differential diagnosis accuracy at brain MRI. Radiology. (2020) 295(3):626–37. 10.1148/radiol.2020190283 32255417 PMC7263320

[55] Guo J Li B. The application of medical artificial intelligence technology in rural areas of developing countries. Health Equity. (2018) 2(1):174–81. 10.1089/heq.2018.0037 30283865 PMC6110188

[56] Meskó B Hetényi G Győrffy Z. Will artificial intelligence solve the human resource crisis in healthcare? BMC Health Serv Res. (2018) 18(1):545. 10.1186/s12913-018-3359-4 30001717 PMC6044098

[57] Callahan TJ Tripodi IJ Hunter LE Baumgartner WA. A framework for automated construction of heterogeneous large-scale biomedical knowledge graphs. Bioinformatics. (2020):2020–04. 10.1101/2020.04.30.071407

[58] Calaprice-Whitty D Galil K Salloum W Zariv A Jimenez B. Improving clinical trial participant prescreening with artificial intelligence (AI): a comparison of the results of AI-assisted vs standard methods in 3 oncology trials. Ther Innov Regul Sci. (2020) 54(1):69–74. 10.1007/s43441-019-00030-4 32008227

[59] Ardizzone E Bonadonna F Gaglio S Marcenò R Nicolini C Ruggiero C Artificial intelligence techniques for cancer treatment planning. Med Inform (Lond). (1988) 13(3):199–210. 10.3109/14639238809010100 3185024

[60] Shaban-Nejad A Michalowski M Buckeridge DL. Health intelligence: how artificial intelligence transforms population and personalized health. NPJ Digit Med. (2018) 1(1):1–2. 10.1038/s41746-018-0058-9 31304332 PMC6550150

[61] Myers TG Ramkumar PN Ricciardi BF Urish KL Kipper J Ketonis C. Artificial intelligence and orthopaedics. J Bone Joint Surg Am. (2020) 102(9):830–40. 10.2106/JBJS.19.01128 32379124 PMC7508289

[62] He J Baxter SL Xu J Xu J Zhou X Zhang K. The practical implementation of artificial intelligence technologies in medicine. Nat Med. (2019) 25(1):30–6. 10.1038/s41591-018-0307-0 30617336 PMC6995276

[63] Momin AA Recinos MA Cioffi G Patil N Soni P Almeida JP Descriptive epidemiology of craniopharyngiomas in the United States. Pituitary. (2021) 24(4):517–22. 10.1007/s11102-021-01127-6 33506438

[64] Young M Delaney A Jurbergs N Pan H Wang F Boop FA Radiotherapy alone for pediatric patients with craniopharyngioma. J Neurooncol. (2022) 156(1):195–204. 10.1007/s11060-021-03908-2 34846639

[65] Lucas JT Jr Faught AM Hsu CY Wilson LJ Guo Y Li Y Pre- and posttherapy risk factors for vasculopathy in pediatric patients with craniopharyngioma treated with surgery and proton radiation therapy. Int J Radiat Oncol Biol Phys. (2022) 113(1):152–60. 10.1016/j.ijrobp.2021.12.172 34990778 PMC9018579

[66] Norris GA Garcia J Hankinson TC Handler M Foreman N Mirsky D Diagnostic accuracy of neuroimaging in pediatric optic chiasm/sellar/suprasellar tumors. Pediatr Blood Cancer. (2019) 66(6):e27680. 10.1002/pbc.27680 30848081

[67] Prince EW Whelan R Mirsky DM Stence N Staulcup S Klimo P Robust deep learning classification of adamantinomatous craniopharyngioma from limited preoperative radiographic images. Sci Rep. (2020) 10(1):16885. 10.1038/s41598-020-73278-8 33037266 PMC7547020

[68] Peng J Kim DD Patel JB Zeng X Huang J Chang K Deep learning-based automatic tumor burden assessment of pediatric high-grade gliomas, medulloblastomas, and other leptomeningeal seeding tumors. Neuro-Oncol. (2022):289. 10.1093/neuonc/noab151 34174070 PMC8804897

[69] Vollmuth P Foltyn M Huang RY Galldiks N Petersen J Isensee F Artificial intelligence (AI)-based decision support improves reproducibility of tumor response assessment in neuro-oncology: an international multi-reader study. Neuro-Oncol. (2023):533. 10.1093/neuonc/noac189 35917833 PMC10013635

[70] Chang SM Wen PY Vogelbaum MA Macdonald DR van den Bent MJ. Response assessment in neuro-oncology (RANO): more than imaging criteria for malignant glioma. Neuro-Oncol Pract. (2015) 2(4):205–9. 10.1093/nop/npv037 PMC6664617 31386074

[71] Hoffman LM Jaimes C Mankad K Mirsky DM Tamrazi B Tinkle CL Response assessment in pediatric craniopharyngioma: recommendations from the response assessment in pediatric neuro-oncology (RAPNO) working group. Neuro-Oncol. (2023) 25:224. 10.1093/neuonc/noac221 36124689 PMC9925711

[72] Lindsay HB Massimino M Avula S Stivaros S Grundy R Metrock K Response assessment in paediatric intracranial ependymoma: recommendations from the response assessment in pediatric neuro-oncology (RAPNO) working group. Lancet Oncol. (2022) 23(8):e393–401. 10.1016/S1470-2045(22)00222-4 35901835

[73] Fangusaro J Witt O Driever PH Bag AK de Blank P Kadom N Response assessment in paediatric low-grade glioma: recommendations from the response assessment in pediatric neuro-oncology (RAPNO) working group. Lancet Oncol. (2020) 21(6):e305–16. 10.1016/S1470-2045(20)30064-4 32502457

[74] Chakrabarty S Abidi SA Mousa M Mokkarala M Hren I Yadav D Integrative imaging informatics for cancer research: workflow automation for neuro-oncology (I3CR-WANO). JCO Clin Cancer Inform. (2023) (7):e2200177. 10.1200/CCI.22.00177 37146265 PMC10281444

[75] Zeineldin RA Karar ME Elshaer Z Coburger J Wirtz CR Burgert O Explainability of deep neural networks for MRI analysis of brain tumors. Int J Comput Assist Radiol Surg. (2022) 17(9):1673–83. 10.1007/s11548-022-02619-x 35460019 PMC9463287

[76] Ak M Toll SA Hein KZ Colen RR Khatua S. Evolving role and translation of radiomics and radiogenomics in adult and pediatric neuro-oncology. Am J Neuroradiol. (2022) 43(6):792–801. 10.3174/ajnr.A7297 34649914 PMC9172943

[77] Nowakowski A Lahijanian Z Panet-Raymond V Siegel PM Petrecca K Maleki F Radiomics as an emerging tool in the management of brain metastases. Neuro Oncol Adv. (2022) 4(1):vdac141. 10.1093/noajnl/vdac141 PMC9583687 36284932

[78] Rudie JD Rauschecker AM Bryan RN Davatzikos C Mohan S. Emerging applications of artificial intelligence in neuro-oncology. Radiology. (2019) 290(3):607–18. 10.1148/radiol.2018181928 30667332 PMC6389268

[79] Vaios EJ Winter SF Shih HA Dietrich J Peters KB Floyd SR Novel mechanisms and future opportunities for the management of radiation necrosis in patients treated for brain metastases in the era of immunotherapy. Cancers (Basel). (2023) 15(9):2432. 10.3390/cancers15092432 37173897 PMC10177360

[80] Stumpo V Staartjes VE Regli L Serra C. Machine learning in pituitary surgery. Acta Neurochir Suppl. (2022) 134:291–301. 10.1007/978-3-030-85292-4_33 34862553

[81] Soldozy S Farzad F Young S Yağmurlu K Norat P Sokolowski J Pituitary tumors in the computational era, exploring novel approaches to diagnosis, and outcome prediction with machine learning. World Neurosurg. (2021) 146:315–21.e1. 10.1016/j.wneu.2020.07.104 32711142

[82] Schilling AT Shah PP Feghali J Jimenez AE Azad TD. A brief history of machine learning in neurosurgery. Acta Neurochir Suppl. (2022) 134:245–50. 10.1007/978-3-030-85292-4_27 34862547

[83] Dai W Zhuang Z Ling H Yang Y Hang C. Systematic review and network meta-analysis assess the comparative efficacy and safety of transsphenoidal surgery for pituitary tumor. Neurosurg Rev. (2021) 44(1):515–27. 10.1007/s10143-020-01240-3 32036504

[84] Titov O Bykanov A Pitskhelauri D. Neurosurgical skills analysis by machine learning models: systematic review. Neurosurg Rev. (2023) 46(1):121. 10.1007/s10143-023-02028-x 37191734

[85] Fu Y Zhang H Morris ED Artificial intelligence in radiation therapy. IEEE Trans Radiat Plasma Med Sci. (2022) 6(2):158–81. 10.1109/TRPMS.2021.3107454 35992632 PMC9385128

[86] Stieler F Yan H Lohr F Wenz F Yin FF. Development of a neuro-fuzzy technique for automated parameter optimization of inverse treatment planning. Radiat Oncol. (2009) 4(1):39. 10.1186/1748-717X-4-39 19781059 PMC2760562

[87] Ebrahimi Zade A Shahabi Haghighi S Soltani M. Deep neural networks for neuro-oncology: towards patient individualized design of chemo-radiation therapy for glioblastoma patients. J Biomed Inform. (2022) 127:104006. 10.1016/j.jbi.2022.104006 35104643

[88] Madhogarhia R Haldar D Bagheri S Radiomics and radiogenomics in pediatric neuro-oncology: a review. Neuro Oncol Adv. (2022) 4(1):vdac083. 10.1093/noajnl/vdac083 PMC9252112 35795472

[89] Longhurst CA Singh K Chopra A Atreja A Brownstein JS. A call for artificial intelligence implementation science centers to evaluate clinical effectiveness. NEJM AI. (2024) 1(8):AIp2400223. 10.1056/AIp2400223

[90] Wright A Phansalkar S Bloomrosen M Best practices in clinical decision support: the case of preventive care reminders. Appl Clin Inform. (2010) 01(03):331–45. 10.4338/ACI-2010-05-RA-0031 PMC3189503 21991299

